# Opportunities to enhance retention on medication for opioid use disorder for adolescents and young adults: results from a qualitative study with medical providers in Philadelphia, PA

**DOI:** 10.1186/s12954-024-01113-8

**Published:** 2024-11-25

**Authors:** Maria Christina Herrera, Kaja Darien, Sarah Wood, Scott E. Hadland, J. Deanna Wilson, Nadia Dowshen

**Affiliations:** 1 Section of Adolescent Medicine, Department of Pediatrics, Yale School of Medicine, 100 Church Street South, Suite A222, New Haven, CT 06520 USA; 2 Division of Adolescent Medicine, Department of General Pediatrics, Children’s Hospital of Philadelphia, Philadelphia, PA USA; 3 Department of Pediatrics, Icahn School of Medicine at Mount Sinai, New York, NY USA; 4 Division of Adolescent and Young Adult Medicine, Mass General for Children, Boston, MA USA; 5 Department of Pediatrics, Harvard Medical School, Boston, MA USA; 6 Department of Family Medicine and Community Health, Perelman School of Medicine at the University of Pennsylvania, Philadelphia, PA USA; 7 Department of Pediatrics, Perelman School of Medicine at the University of Pennsylvania, Philadelphia, PA USA

**Keywords:** Adolescent and young adult, MOUD, Retention, Qualitative interviews, Chart-stimulated recall

## Abstract

**Background:**

Medications for opioid use disorder (MOUD) are under-prescribed to adolescents and young adults (AYA). Few published studies have explored challenges to and opportunities to enhance continuous provision of MOUD for AYA. Our report focuses on this emergent theme that was identified as part of a larger qualitative study.

**Methods:**

We purposively sampled and enrolled medical providers who prescribed MOUD to AYA. Semi-structured individual interviews using chart-stimulated recall explored barriers and facilitators to MOUD retention. We used modified grounded theory in our qualitative analysis, with double coding of interviews.

**Results:**

Barriers to retention on MOUD included patient-level (i.e., return to substance use) and system-level factors (i.e., cost, delayed receipt, pharmacy challenges, and in-person visit requirements). Facilitators included patient-level (i.e., motivation, support networks) and system-level factors (i.e., telehealth access, availability of certified recovery specialists).

**Conclusions:**

Our study is the first to look at *retention* for this key age group, setting it apart from the existing body of literature that looks at medication *initiation*. Our findings confirm that significant systemic barriers exist to AYA patients’ retention on MOUD. Further research is needed to develop interventions that facilitate continuous delivery of high-quality care among this key population.

## Introduction

Opioid overdoses are a leading cause of death in adolescents and young adults (AYA) internationally [1]. For AYA with an opioid use disorder (OUD), the Society for Adolescent Health and Medicine and other major medical organizations recommend timely treatment with medications for opioid use disorder (MOUD) [2–4]. However, in a recent analysis of national addiction treatment program data, younger age (18–29 years) was one of the strongest risk factors for MOUD discontinuation by 6 months [5]. Yet no studies have investigated specific barriers and facilitators to MOUD retention for adolescents with OUD. OUD is a chronic medical condition benefitting from longitudinal treatment, including use of MOUD for at least 2–3 months and in many cases, extending over several years [6]. Extant work describes the lack of access to treatment initiation resources for young people with OUD [7–9]. However, no studies have focused on contextualizing the barriers and facilitators to MOUD retention for this age group. Understanding these barriers and facilitators will be critical to increase MOUD retention for this population at high risk of death by overdose, which can be exacerbated when the healthcare system fails to meet their unique needs. To address this gap, we conducted a qualitative study with providers examining the barriers and facilitators to MOUD retention in patients with history of MOUD receipt.

## Methods

We conducted semi-structured individual interviews in Philadelphia from December 2021 to April 2022 as part of a study about integrating addiction treatment and HIV prevention among AYA [10]. The theoretical framework used to develop the interview guide was the Practical Robust Implementation Sustainability Model (PRISM) [11]. Participation was limited to physicians, physician assistants (PAs), or nurse practitioners (NPs) who had prescribed either MOUD or HIV pre-exposure prophylaxis (PrEP) to patients aged 16 to 29 years. All participants completed a verbal informed consent process with study staff that detailed the study purpose, protocol, and potential risks and benefits. Recruitment ended after reaching thematic saturation as determined by the primary investigator (M.C.H) and research assistant (K.D). All transcripts were coded independently by a team of 2 experienced qualitative investigators. Consistent with the methods of the parent study, the 2 coders met to discuss and reconcile their individually assigned codes using a κ coefficient threshold of 0.8 to ensure the rigor of interrater reliability. Exemplary quotes were identified. Interviews were analyzed using grounded theory as well as iterative, inductive techniques reliant on content analysis to identify key themes. While the interview guide primarily explored barriers and facilitators to MOUD and PrEP integration, this brief communication focuses on results related to retention in MOUD care, which was an emergent theme identified in interviews after completion of data collection. Key study findings are presented following the Standards for Reporting Qualitative Research [12]. This study was approved by the Children’s Hospital of Philadelphia Institutional Review Board.

## Results

Participants (n = 19) were comprised of physicians (63%), nurse practitioners (21%) and physician assistants (16%), and 90% had prescribed MOUD to AYA. Over half the sample (63%) identified as cis-gender female with the remainder identifying as cis-gender male. The majority of our sample reported either less than 5 or 5–10 years since completion of their professional training (47% and 32% respectively). Key barriers included system-level and patient-level factors (Table 1). At the system-level, cost was reported to be a barrier to retention in MOUD care given out-of-pocket expenses associated with certain MOUD options depending on insurance coverage (Participant #5). The need to switch young people to different formulations was also discussed by providers as decisions they made jointly with patients for cost-savings (Participant #10). One participant, who informed patients ahead of time how changes in insurance could impact the cost of MOUD, discussed the impact of public versus private insurance status on cost of MOUD and how private insurance would be associated with greater costs to the patient (Participant #15). In addition to cost, providers also reported delays in patient receipt of MOUD depending on requirement of insurance prior authorizations and timely arrival of long-acting injectable forms of MOUD in clinic for administration during monthly scheduled visits.

**Table 1:** Barriers to MOUD retention and exemplar quotes from medical providers for AYA with OUD

Patient-level Barriers
*Return to Substance Use* *Participant* #2: “It’s been complicated with her. She had one relapse while she was on the suboxone. She had come to us on suboxone, and so she was getting it on the streets. Technically, it was like a restart. Then we did kind of lose her to care for a couple of months when she did have a fairly significant relapse on heroin, ended up in the hospital, needed Narcan. And then came back to us after that, reached out to our clinic. And we then restarted her suboxone.” *Participant* #3: “This is a guy who is very conflicted and hates having to make a decision every day about whether to use illegal drugs or not. And Sublocade felt right to him that he would not have to make that decision every day. He had been thinking about it for a long time. We actually had it in our fridge with his name on it, because he had said he wanted it months ago but then changed his mind when he admitted that he had slipped up and started heavily using Xanax again and that he had totaled his car and that he was now homeless, because his girlfriend had kicked him out. There was a lot of drama around the relapse with the Xanax.”
System-level Barriers
*Cost* *Participant* #10: “He pays out-of-pocket for his medications. For patients where cost is an issue, tablet form of generic suboxone is ideal. He was on films, but for cost we switched to tablets. Also he had been—I had tried to dose him higher in the beginning, he was up to 20 mg, not really 24, partly because of cost. And he stabilized at 16 mg, he takes it one time a day and he’s able to afford that.” *Participant* #12: “There are definitely people who can’t get the medications, or can’t afford it, or it’s a huge burden for them to pay for their medication. I would say for vivitrol, that’s the biggest thing. That’s a huge reason why a good portion of my patients want vivitrol but can’t get it because of the cost. Suboxone, I think, tends to be easier and more covered, but there definitely are people that I have that are paying up to $50 a week. And if they really understand how important it is, they’ll prioritize that. But that money is kind of getting pulled from childcare, or food, or educational, saving goals they have… rent.” *Participant* #15: “We help patients get on state Medicaid for the most part, so most of them don’t have insurance issues. We have good relationships with other pharmacies where if they’re on buprenorphine/Naloxone films or tablets they’ll like float costs for a month if they lose insurance. That’s not something we can do with Sublocade, and I do kind of let people know that if you get a job and you get private insurance, it’s going to cost more, or if you lose insurance it’s not something that we’re going to really be able to float.”
*Delayed Receipt* *Participant* #1: Usually when someone decides they want to go onto Sublocade, we say, okay, that sounds great. We will order it. We will see if your insurance covers it. We will get it shipped here to the office, and we can have it here as soon as two weeks. It’s almost never the case that someone comes in for the medication and it’s sitting there waiting for them *Participant* #13: “A big struggle for us was prior authorizations. We would prescribe for the first time for a patient, they’d never been on a medication before and then they would go to the pharmacy, they’re ready, we’ve got them at the perfect level of just slightly withdrawing and then the med needs a prior auth and then you have to wait to try and get it approved.”
*Pharmacy Challenges* *Participant* #10: “We have so many problems with pharmacies, countless problems. And this particular pharmacy, yes, has said, we don’t feel comfortable doing this. And I call them and say, she, this patient, doesn’t have a driver’s license, so transportation to pharmacy was a barrier and she can walk to this pharmacy. And it’s her only option and therefore, I need them to help. And so they continued to dispense, but yes, they cause—a lot of times they say, oh, we don’t want to fill the meds because we think she’s using because she’s buying needles when she’s picking up her suboxone. And we say, okay, well great, she’s using clean needles and she’s—maybe she’s giving them away, we don’t know. And it’s like, either way, that’s our job. It’s definitely a barrier.” *Participant* #11: “Pharmacies have what they call, caps, where they won’t take on any more patients because they already—they’re saying that they are capped by the pharmaceutical companies on how many patients they can have that are filling suboxone or generic formulations, apparently it doesn’t matter. I ran into an issue today where they said that their patient lived outside of the town limits.” *Participant* #14: “There are a lot of community pharmacies where they don’t—cutting films is a big part of prescribing and if you have to micro-dose somebody or if you have to adjust their dose so that they feel comfortable, cutting films is a part of that, that’s how we tell people to take their medications. And there are plenty of community pharmacies who actually refuse to fill a prescription because we wrote anything about cutting a film on the prescription itself. So we have to send over these prescriptions for films and then write out detailed instructions for the patients, which I don’t think is the safest way to prescribe, it’s much better to just have it all consistent across the board. I think that would be another barrier, because there’s a lot of pharmacies that don’t feel comfortable giving a patient instructions to manipulate the films in any way. Even though cutting the films is a standard part of prescribing.” *Participant* #16: “I also have had multiple patients who have issues with their home pharmacies regularly causing them trouble with their buprenorphine scripts, which I find very annoying. Honestly, our patients lead pretty chaotic lives and so it will happen with some regularity that a patient will—not usually the same patient but a patient at some point will write in and be like, I was on the bus on the way home and I got off the bus and I got mugged and they took all my buprenorphine, which probably did happen. They’re not making it up. They live tough lives. But then, even if I write a refill script and the insurance company might even be willing to authorize the override, then their pharmacy will be like, well, this is shady, we’re not going to refill their scripts early. And my philosophy on that is like, who cares? If there’s more buprenorphine out in the world, so what.”
*Requirement for In-Office Visits* *Participant* #10: “I know for the last patient we were discussing, in terms of his treatment, for him telehealth treatment was really important because for him finances and getting to a treatment and maintaining his job, that was really important to him. It’s difficult for patients to do both, right? We get them functionally back to work and then it’s a lot to ask them to drive and go far away to a treatment.”

Participants also discussed system-level barriers at the pharmacy, noting that patients faced a wide array of challenges such as refusal of a pharmacist to dispense the prescribed dose if perceived as too high or if the pharmacist harbored stigma against suspected continued substance use (Participant #8). Some participants could not receive their MOUD prescription at their preferred pharmacy because of “caps” wherein the pharmacist reported being “capped by the pharmaceutical companies on how many patients they can have that are filling suboxone or generic formulations” or because the patient lived “outside the town limits” (Participant #11). Additionally, some patients in need of an emergency bridge prescription due to MOUD being lost/stolen were turned away by pharmacies that refused to fill MOUD prescriptions early even when covered by insurance (Participant #16). Lastly, the need to present physically for in-person office visits posed a burden for some patients due to lack of transportation or conflicting employment obligations which interfered with their ability to continue MOUD (Participant #10). At the patient-level, history of return to substance use influenced MOUD retention for AYA, including return to use of both opioids and other substances such as benzodiazepines (Participant #3).

With respect to facilitators (Table 2), at the system-level, the ability to offer telehealth was cited as a factor that enabled MOUD retention (Participant #18), as well as the linkage of patients to certified recovery specialists (CRS) (Participant #5). At the patient-level, participants emphasized the support of a patient’s social network as an integral facilitator to MOUD retention. Patients who had parental or other familial involvement, who were parents themselves or had supportive partners were noted to be more likely to continue on MOUD. Motivation as it relates to employment attainment was also a facilitator to being retained in MOUD care (Participant #7).

**Table 2:** Facilitators to MOUD retention and exemplar quotes from medical for AYA with OUD

Patient-level Facilitators
*Patient Motivation* #7: “Well, I think he is just kind of a pretty—he’s just a very motivated person and he has stable housing and he has a job. He’s just kind of this exceptional and very sweet and pleasant, 25-year-old. So he might not really represent everyone, but not necessarily because he comes from a place of privilege, just because he’s this special person, I think.” #10: “So she is very aware of the problems that her opioid use caused in her life and she was incarcerated for a year. She’s very determined not to have those problems come back in her life. She now has a 3-year-old son, which I guess the timeline would be right before incarceration she had the child. So she’s very driven by that. She does not want to use opioids anymore. She’s very, very adamant about that.”
*Supportive Social Networks* *#7: “H*e has a fiancé, who is very supportive. And she doesn’t use substances and they seem to have a very sweet relationship. One of the nice things about telehealth and getting a view into someone’s home is that there have been a few visits where she’s been around and has walked by and said, hi. And their interactions are very sweet and it just seems like he—yeah, he’s very supported.” #9: “A big thing with this patient is that his mother has been very involved in his care. He lives with his parents. I speak to his mother between every appointment, kind of get her input on everything. We discuss what’s going on and she feels he’s doing very well, making a lot of improvements. And he also recognizes other positive changes in his life, not related to drug use. He’s going to school. He’s going to classes more. He’s got a new girlfriend. His relationship with his parents is doing well.”
System-level Facilitators
*Availability of Navigation Staff* #6: “It turns out to no one’s surprise, that many members of our staff have either been personally affected or have family that’s been affected by opioids, and they were so excited that we were offering MOUD and they have raised their hand to say, I want to be the go-to person. I have a go-to nurse. I have a go-to patient service associate. My practice lead I think has had a family member affected by this, so he has been like, you tell me what you need and I’ll make it happen. So it really is key to rope in folks in the office who are willing to help and to train them up and get them to understand the unique issues.” #19: “I mean definitely our certified recovery specialist has been indispensable. She really follows up with the patients and is like, hey, remember your appointment. You’re coming, right? You’re coming.”
*Telehealth Access* #11: “Allowing more telehealth has been so beneficial for low barrier buprenorphine.” #18: “I think really for a lot of patients it’s just the ability to do the visit from home and not have to go anywhere. That’s a big selling point.”

## Discussion

Increasing access to MOUD for AYA is a critical means toward preventing the ongoing loss of young lives to fatal opioid overdose [13].Among medical providers caring for AYA with OUD there were multiple barriers identified to retaining patients in care and ensuring that they can receive life-saving, recommended medications to treat OUD on a continuous basis [14, 15]. Our findings provide key insights into the daily experience of providers and their patients who are fighting to access MOUD and areas for future intervention development. As our findings show, young people face stigma when trying to obtain their prescriptions, which has been described as a barrier among older adults as well [16]. We recommend collaboration with pharmacy colleagues to ensure universal, comprehensive substance use disorder treatment and harm reduction education. Since this study was conducted the Consolidated Appropriations Act of 2023 [17] was introduced to repeal the special waiver and mandatory training hours for prescribing buprenorphine to patients with OUD. While this law holds immense potential to expand MOUD access, we cannot lose sight of the infrastructure needed to support MOUD retention. For example, advocacy to overcome insurance barriers is critical for this population, who are often facing transitions from parental to personal insurance and navigating employer-based insurance plans for the first time [18]. Medical providers should discuss common insurance-level barriers with AYA and offer contingency planning if their prescriptions are not dispensed.

With the limited geographic capture of our study, a future extension of this study could explore facilitators and barriers among young people living in more rural parts of the country. Another important limitation is that this study does not include the patient perspective, which we hope to elicit in future studies. Although this study focused on a single regional community of medical providers who treat AYA with OUD where potential biases may exist, there are widely applicable findings. Confidential, accessible services will be critical for young people who use drugs so they may remain engaged in their other life priorities. Given the importance of remaining on MOUD for long periods of time to achieve remission (i.e., months to years), our work is impactful in that it is the first to look at *retention* for this key age group, setting it apart from the existing body of literature that looks at medication *initiation*. Conceptualizing care models that include key supporters such as family, social network members, and CRS will also be important to leverage strengths noted by providers in this study that are pivotal for youth retention on MOUD.

## Data Availability

No datasets were generated or analysed during the current study.

## Abbreviations

AYA: Adolescents and Young Adults
OUD: Opioid Use Disorder
MOUD: Medications for Opioid Use Disorder
HIV: Human Immunodeficiency Virus
PrEP: Pre-exposure Prophylaxis

## References

[1] Baumgartner JC, Gumas ED, Gunja MZ. Too many lives lost: overdose mortality rates and policy solutions | Commonwealth Fund. To the Point (blog), Commonwealth Fund.

[2] Waldron LJ, Dakessian S. Medication for adolescents and young adults with opioid use disorder. The society for adolescent health and medicine. J Adolescent Health. 2021;68(3):632–6. 10.1016/j.jadohealth.2020.12.129 PMC7902443 33485735

[3] Ryan SA, Gonzalez PK, Patrick SW, Quigley J, Siqueira L, Walker LR. Committee on substance use and prevention. Medication-assisted treatment of adolescents with opioid use disorders. Pediatrics. 2016;138(3):e20161893. 27550978 10.1542/peds.2016-1893

[4] The ASAM National Practice Guideline for the Treatment of Opioid Use Disorder: 2020 Focused Update. J Addict Med. 2020 Mar/Apr;14(2S Suppl 1):1–91. 10.1097/ADM.0000000000000633 32511106

[5] Krawczyk N, Williams AR, Saloner B, Cerdá M. Who stays in medication treatment for opioid use disorder? A national study of outpatient specialty treatment settings. J Subst Abuse Treat. 2021;126: 108329. 34116820 10.1016/j.jsat.2021.108329 PMC8197774

[6] Sordo L, Barrio G, Bravo MJ, Indave BI, Degenhardt L, Wiessing L, Ferri M, Pastor-Barriuso R. Mortality risk during and after opioid substitution treatment: systematic review and meta-analysis of cohort studies. BMJ. 2017;26(357): j1550. 10.1136/bmj.j1550 PMC5421454 28446428

[7] King C, Beetham T, Smith N, et al. Treatments used among adolescent residential addiction treatment facilities in the US, 2022. JAMA. 2023;329(22):1983–5. 37314282 10.1001/jama.2023.6266 PMC10265296

[8] Terranella A, Guy GP, Mikosz C. Buprenorphine dispensing among youth aged ≤19 years in the United States: 2015–2020. Pediatrics. 2023;151(2): e2022058755. 36691760 10.1542/peds.2022-058755 PMC10142390

[9] Alinsky RH, Hadland SE, Matson PA, Cerda M, Saloner B. Adolescent-serving addiction treatment facilities in the united states and the availability of medications for opioid use disorder. J Adolesc Health. 2020;67(4):542–9. 32336560 10.1016/j.jadohealth.2020.03.005 PMC7508760

[10] Herrera MC, et al. A qualitative study of barriers and facilitators to integrating medications for opioid use disorder and HIV preexposure prophylaxis for adolescents and young adults. J Addict Med. 2023. 10.1097/ADM.0000000000001195. 37934522 10.1097/ADM.0000000000001195 PMC10740380

[11] Feldstein AC, Glasgow RE. A practical, robust implementation and sustainability model (PRISM) for integrating research findings into practice. Jt Comm J Qual Patient Saf. 2008;34(4):228–43. 18468362 10.1016/s1553-7250(08)34030-6

[12] O’Brien BC, Harris IB, Beckman TJ, Reed DA, Cook DA. Standards for reporting qualitative research: a synthesis of recommendations. Acad Med. 2014;89(9):1245–51. 24979285 10.1097/ACM.0000000000000388

[13] Tanz LJ, et al. Drug overdose deaths among persons aged 10–19 years-United States, July 2019-December 2021. Morbid Mortal Weekly Rep. 2022;71(50):1576. 10.15585/mmwr.mm7150a2 PMC9762908 36520659

[14] Camenga DR, Hammer LD. The Committee on Substance Use and Prevention, and Committee on Child Health Financing; Improving Substance Use Prevention, Assessment, and Treatment Financing to Enhance Equity and Improve Outcomes Among Children, Adolescents, and Young Adults. Pediatrics. 2022;150(1):e2022057992. 10.1542/peds.2022-057992. 35757960 10.1542/peds.2022-057992

[15] Hadland SE, et al. Receipt of timely addiction treatment and association of early medication treatment with retention in care among youths with opioid use disorder. JAMA Pediatr. 2018;172(11):1029–37. 30208470 10.1001/jamapediatrics.2018.2143 PMC6218311

[16] Volkow ND. Stigma and the toll of addiction. N Engl J Med. 2020;382(14):1289–90. 32242351 10.1056/NEJMp1917360

[17] *Consolidated Appropriations Act*.; 2023. Accessed August 30, 2024. https://www.appropriations.senate.gov/imo/media/doc/JRQ121922.PDF

[18] Spencer DL, McManus M, Call KT, Turner J, Harwood C, White P, Alarcon G. Health care coverage and access among children, adolescents, and young adults, 2010–2016: Implications for future health reforms. J Adolesc Health. 2018;62(6):667–73. 29599046 10.1016/j.jadohealth.2017.12.012 PMC5964030

